# Context and Individual Characteristics Modulate the Association between Oxytocin Receptor Gene Polymorphism and Social Behavior in Border Collies

**DOI:** 10.3389/fpsyg.2017.02232

**Published:** 2017-12-19

**Authors:** Borbála Turcsán, Friederike Range, Zsolt Rónai, Dóra Koller, Zsófia Virányi

**Affiliations:** ^1^Comparative Cognition and Wolf Science Center, Messerli Research Institute, University of Veterinary Medicine Vienna, Medical University of Vienna, University of Vienna, Vienna, Austria; ^2^Institute of Cognitive Neuroscience and Psychology, Research Centre for Natural Sciences, Hungarian Academy of Sciences, Budapest, Hungary; ^3^Department of Medical Chemistry, Molecular Biology and Pathobiochemistry, Semmelweis University, Budapest, Hungary

**Keywords:** oxytocin receptor gene, dog, greeting behavior, stress, individual differences, contextual differences

## Abstract

Recent studies suggest that the relationship between endogenous oxytocin and social affiliative behavior can be critically moderated by contextual and individual factors in humans. While oxytocin has been shown to influence human-directed affiliative behaviors in dogs, no study investigated yet how such factors moderate these effects. Our study aimed to investigate whether the context and the dogs’ individual characteristics moderate the associations between the social affiliative (greeting) behavior and four single-nucleotide polymorphisms (SNPs) of the oxytocin receptor (*OXTR*) gene. We recorded the greeting behavior in three contexts: (1) when the dog first met an unfamiliar experimenter, (2) during a separation from the owner, and (3) after the experimenter approached the dog in a threatening manner. In the latter two contexts (during separation and after threatening), we categorized the dogs into stressed and non-stressed groups based on their behavior in the preceding situations. In line with previous studies, we found that polymorphisms in the *OXTR* gene are related to the greeting behavior of dogs. However, we also showed that the analyzed SNPs were associated with greeting in different contexts and in different individuals, suggesting that the four SNPs might be related to different functions of the oxytocin system. The -213A/G was associated with greeting only when the dog had no prior negative experience with the experimenter. The rs8679682 was found in association with greeting in all three contexts but these associations were significant only in non-stressed dogs. The -94T/C was associated with greeting only when the dog was stressed and had an interaction with the sex of the dog. The -74C/G SNP was associated with greeting only when the dog was stressed during separation and also had a sex interaction. Taken together, our results suggest that, similarly to humans, the effects of oxytocin on the dogs’ social behavior are not universal, but constrained by features of situations and individuals. Understanding these constraints helps further clarify how oxytocin mediates social behavior which, in the long run, could improve the application of oxytocin in pharmacotherapy.

## Introduction

Encountering an unfamiliar individual can always carry some risk. Initiating interactions (especially affiliative or cooperative interactions) with a stranger without knowing his/her attitude and intentions could lead to unpleasant or even dangerous experiences. Research on humans and non-human animals showed that the oxytocin system plays a prominent role in evaluating the potential benefits and risks of social encounters, and thus, modulating the willingness to approach and engage another individual in social interactions (Young et al., 2001; Depue and Morrone-Strupinsky, 2005; Lim and Young, 2006). Various mechanisms have been proposed to explain this influence of oxytocin, a peptide hormone produced in the hypothalamus. Humans’ social interactions with others are associated with greater amygdala activation than non-social interactions (Eiji Nawa et al., 2008), and oxytocin is thought to down-regulate this activation (Tost et al., 2010; Kumsta and Heinrichs, 2013). On the other hand, oxytocin has been proposed to link approach behavior to the rewarding experience of social interactions (Insel, 2003; Campbell, 2008), and to facilitate the categorization of others as in-group members (De Dreu, 2012), thereby promoting positive interactions during social encounters.

Oxytocin shows remarkable evolutionary preservation in structure and function (Donaldson and Young, 2008). Therefore, it is an especially interesting question how it contributes to regulating social behavior in today societies where meeting and interacting with strangers happen extremely frequently. Humans share this open social environment with dogs, and both species, indeed, seem to be strongly motivated to engage in social interactions (Over, 2016; vonHoldt et al., 2017). Despite their genetically based gregariousness, the social behavior of dogs toward humans is not at all uniform. There is considerable variation in this phenotype not only across but also within breeds (Mehrkam and Wynne, 2014; Persson et al., 2015), and recent studies have showed that there is a significant genetic basis for this variation (Persson et al., 2015, 2016). In particular, genetic variation in the oxytocin system has been put forward as a prominent candidate to account for differences in human-directed social behavior, including interactions with strangers (Beetz et al., 2012).

Polymorphisms of the oxytocin receptor (*OXTR*) gene have been most investigated so far, and have indeed been found to be associated with behavioral variation that humans and dogs show when interacting with strangers (see Kumsta and Heinrichs, 2013; Li et al., 2015 for human reviews). Variations in the *OXTR* gene sequence can influence the location, density, distribution pattern, and functioning of the oxytocin receptors (Young et al., 2001; Donaldson and Young, 2008; MacDonald and MacDonald, 2010; Meyer-Lindenberg et al., 2011), and animal research has demonstrated that differences in the neural distribution and expression of the receptors often lead to individual differences in social behavior (Insel and Shapiro, 1992). Taken together, genetic variation of the *OXTR* gene can alter receptor density, affinity, or function in specific brain regions, thereby moderating the subjects’ sensitivity to oxytocin, and in turn their behavior.

The behavioral associations of *OXTR* variation are far from uniform, however. In dogs, Kis et al. (2014) found for instance that two out of three single-nucleotide polymorphisms (SNPs) of the *OXTR* gene had opposite associations with stranger-directed behavior in two breeds. The -213AG polymorphism was the only one that was similarly associated with proximity seeking both in Border collies and in German shepherds: dogs carrying the G allele approached and followed a stranger less than AA dogs. The two other polymorphisms investigated (rs8679684 and 19208A/G) were associated with friendliness, but differently in the two breeds: in German shepherds carriers of the A allele for both SNPs were more friendly, whereas in Border collies individuals carrying the A allele were less friendly (Kis et al., 2014). Furthermore, while Kubinyi et al. (2017) reported findings on Siberian huskies similar to the above results in Border collies, they failed to find associations between the same three SNPs and the greeting behavior of Border collies. Only in huskies the G/G homozygotes on the 19208A/G SNP were faster and more persistent in greeting an unfamiliar human than dogs carrying the rare A allele.

The inconsistent findings of both human and non-human studies seem to confirm concerns questioning the explanatory power of individual SNPs (Benjamin et al., 2012). Others argue, however, that much of this inconsistence comes about because the effects of oxytocin depend on the context and on the characteristics of the individual (sex, personality traits, etc.) (Bartz et al., 2011). As an example, behavioral associations of *OXTR* polymorphisms have been shown to depend on the social environment the subjects lived in, as well as on the context their behavior was tested in (Kim et al., 2010; Chen et al., 2011). Kim et al. (2010) showed that in humans the G allele of the rs53576 *OXTR* SNP, relative to the AA genotype, was associated with higher emotional support seeking in American subjects but not in Korean subjects, and even in American subjects only in periods of distress. This finding they explain by the difference that in American culture it is normative to seek emotional support in times of distress but not in Korean culture, and suggest that psychological distress and culture are important moderators that shape behavioral outcomes associated with *OXTR* genotypes. In concert with this finding, Chen et al. (2011) showed that individuals carrying at least one copy of the G allele of the same SNP could benefit more from receiving social support than AA individuals. Subjects with GG or AG genotype showed significantly lower cortisol and subjective stress rating relative to the AA genotype, but only when they received social support while preparing for a stressful encounter with strangers. These results suggest that experiencing stress is an important modulator of the behavioral effects of the genetic variation of *OXTR*. More specifically, when an individual perceives a situation stressful, certain *OXTR* genotypes may be more likely to promote approach and affiliation (as a form of social support seeking, and thereby buffering the stress response), whereas the positive effect of these genotypes in support seeking may not be evident in times and contexts when the individual is not experiencing stress.

In the current study we set out to investigate whether and to what extent the context of the situation and the dogs’ experiencing stress moderate the associations between pet dogs’ social approach and affiliation (greeting behavior) to an unfamiliar human and polymorphisms in their *OXTR* gene. Greeting behavior (describing how the dog approaches and interacts with a friendly but unfamiliar experimenter) is frequently assessed in dog studies, and it is related to the sociability personality trait of the individuals (Svartberg and Forkman, 2002). Studies have also showed that the dogs’ greeting behavior (and sociability in general) has a genetic background (e.g., van der Waaij et al., 2008; Persson et al., 2015), although its heritability might be breed-dependent (Héjjas et al., 2009; Wan et al., 2013). In the current study, the dogs’ greeting behavior was tested in three contexts: when the dog first met the experimenter, when the dog was separated from the owner, and after the experimenter approached the dog in a threatening manner. In order to test for moderating effect of stress, in each of the second and the third contexts, based on their reaction to separation and to a social threat, respectively, we categorized the dogs into two groups: (1) dogs either stressed or not by being separated from the owner when in an unfamiliar place, and (2) dogs either stressed (showing an overt avoidant or aggressive reaction) or not (reacting in a friendly way or passively) by the experimenter, when she approached them in a threatening way. Both the separation from the owner (especially when at the same time also facing a stranger) and the threatening approach had been found to evoke a stress response in dogs both at a behavioral and a physiological level (i.e., increased heart rate and heart rate variability in separation: Palestrini et al., 2005; Maros et al., 2008; and increased cortisol concentrations after the threatening approach: Horváth et al., 2008). Beyond this, however, dogs show a rather diverse sensitivity for both situations (Topál et al., 1998; Vas et al., 2008) that are differently stressful for the different individuals. Gácsi et al. (2013) found that dogs which were behaviorally reactive in these two situations (measured by vocalization) showed increased heart rate/heart rate variability after the test, but no significant cardiac response was found in non-reactive dogs. Due to this variation, these two situations seemed suitable to investigate whether the dogs’ stress reactivity interacts with their *OXTR* genotype in affecting behavior during social encounters. Furthermore, we investigated whether the sex and age of the dogs moderated the associations between *OXTR* polymorphisms and greeting behavior. We chose a single breed for the analyses because there are marked differences regarding *OXTR* variations between different breeds (Bence et al., 2017); thus, the genetic constitutions of the breeds could overshadow the effect of one candidate gene, and because previous studies found breed differences both in social behavior and in the associations between *OXTR* polymorphisms and social behavior (Kis et al., 2014; Kovács et al., 2016; Kubinyi et al., 2017).

## Materials and Methods

### Ethics Statement

This study was approved by the institutional ethics and animal welfare committee at the University of Veterinary Medicine Vienna (Approval numbers: 09/04/97/2012, 04/05/97/2012, 09/10/97/2012) in accordance with Good Scientific Practice guidelines and national legislation^1^. The owners participated on a voluntary basis and they all signed an informed consent form before beginning the experiment.

### Subjects

Altogether, 217 purebred Border collies, recruited from the Clever Dog Lab database in Vienna, participated in a behavior test battery and were genotyped for *OXTR* polymorphisms. In the current analyses, we excluded dogs younger than 10 months and dogs with missing genotype data. The final sample consisted of *N* = 173 dogs, 72 (41.6%) males and 101 females, and their mean age (±SD) was 3.87 ± 3.02. From this sample, due to technical reasons, two dogs had missing values for the *Greeting during first encounter*, four dogs for the *Greeting during separation*, and one dog for the *Greeting after threatening approach* context (please see below for descriptions of test contexts).

### Procedure

#### Phenotyping

The test was conducted in an experimental room of the Clever Dog Lab (5 m × 6 m), and dogs were allowed to explore the room prior to the test. The dogs participated in three situations, all three were presented on the same day with ca. 20 min between them. The order of the test contexts was the same for all subjects, and the experimenter was the same in all three contexts.

(1) Greeting during first encounter (see Héjjas et al., 2009; Wan et al., 2013; Kis et al., 2014).

The owner held the dog on a loose leash in the middle of the test room and was instructed not to talk to or interact with the dog. The experimenter (unfamiliar to the dog) entered the room, verbally greeted the owner and the dog, and then approached the dog in a friendly manner (walking at a normal pace, looking in the direction of the dog, and smiling). The experimenter stopped 1.5 m away from the dog.

• If the dog approached the experimenter and showed “friendly” behaviors (moving toward her and tail wagging) or remained neutral (did not move away from the owner, no tail wagging, and no aggression), then the experimenter petted it while continuously speaking in a friendly way.
• If the dog showed fearful/stress behaviors (avoiding eye contact, low body posture, and low ear position), or actively avoided the experimenter, then she crouched down and called the dog. If the dog approached in a non-aggressive manner, the experimenter petted it while continuously speaking in a friendly way. If the dog did not respond, the experimenter talked continuously to the dog in a friendly manner for 10 s and then terminated the test.
• If the dog growled or barked at the experimenter, then she talked continuously to the dog in a friendly manner for 10 s while avoiding eye contact, and then terminated the test.

(2) Greeting during separation.

The dog was unleashed and left alone in the experimental room. After 1 min, the experimenter entered the room, stood next to the door for 5 s without interacting with the dog, and then followed the protocol of the “*Greeting during first encounter*” test context.

(3) Greeting after threatening approach.

The protocol of the threatening approach was similar to the procedure described in Vas et al. (2005). The owner held the leash of the dog and he/she was not allowed to talk or interact with the dog. The experimenter called the dog’s name, and then approached the dog slowly and haltingly, with a slightly bent upper body while staring steadily into the eyes of the dog. The approach was terminated if (a) the experimenter reached the dog’s position, (b) the dog approached her in a non-aggressive manner, (c) the dog moved away and hid behind the owner, or (d) the dog reacted aggressively (e.g., excessive growling or barking, snapping, or attacking).

After the threatening approach was terminated, the experimenter stepped a few steps away from the dog while the owner unleashed the dog, crouched down, called the dog in a friendly manner, and then followed the protocol of the “*Greeting during first encounter*” test.

#### Coded Variables

The dogs’ behavior was recorded by four cameras located in the corners of the testing room and the recordings were analyzed at a later date. The same three variables were coded in all three greeting contexts: if, when, and how the dog approached the experimenter, how much enthusiasm the dog showed during the greeting, and if and how much the dog wagged its tail (for the definitions of the variables, see **Table 1**). We created a scale for each context by taking the mean of these three variables. The three scales showed good internal consistency (Cronbach’s α > 0.6, **Table 2**). To assess the inter-observer reliability of the scales, a subset of 40 videos was coded twice by two of three independent coders. The intraclass correlation coefficients (ICC 1,*k*, absolute agreement; Weir, 2005) calculated between the coders were >0.8 for all three scales, indicating good inter-observer reliability (**Table 2**).

**Table 1:** Variables coded in the test: approach, enthusiasm, and tail wagging.

Situation(s)	Variable	Definition
	Approach	When E approaches, the dog …
		0: does not approach her on its own;
		1: approaches when called;
		2: approaches hesitatingly or after a while;
		3: approaches immediately without calling.
	Enthusiasm	The dog …
(1) Greeting during first encounter		0: is not interested, avoids interacting with E (i.e., turns away or withdraws);
(2) Greeting during separation		1: behaves passively, does not elicit interaction (i.e., stays in one place, may sniff around a bit);
(3) Greeting after threatening approach		2: behaves friendly (i.e., approaches the E, may cuddle, jump or lick once);
		3: is very excited/enthusiastic with intensive searching for contact (i.e., rushes to E, cuddles, jumps up or licks her, tries to stay close and in physical contact with E).
	Tail wagging	The dog …
		0: shows no or very little tail wagging;
		1.5: wags its tail intermittently;
		3: wags its tail continuously
Separation	Stress signals	During the 1-min long separation period the dog …
		0: does not show any (detectable) stress signals;
		1: shows signs of stress, including vocalization, pacing, yawning, lip licking, salivation, stretching, self-grooming, shaking, or scratching the door
Threatening approach	Reaction to threat	Behavior shown just before the test is terminated:
		0: the dog approaches E with tail wagging or remains passive (i.e., no approach and no avoidance, may wag tail intermittently);
		1: the dog hides behind the owner or moves away from the E (with low tail and ear position) or shows signs of aggression (i.e., barking, growling, snapping, or lunging toward E).


*These three variables were coded in each of the three greeting contexts, and for each context they were combined into a scale. E, experimenter.*

**Table 2:** Reliability measures of the three greeting scales.

	Internal consistency	Inter-observer reliability	
		
Situation	Cronbach’s α	ICC	*F*-test
Greeting during first encounter	0.727	0.878	*F* = 8.211, *p* < 0.001
Greeting during separation	0.758	0.834	*F* = 6.028, *p* < 0.001
Greeting after threatening approach	0.675	0.868	*F* = 7.549, *p* < 0.001


*ICC, intraclass correlation coefficient.*

Additionally, we also coded how the dogs reacted to separation from their owner and to being approached in a threatening way by the experimenter. During separation, we coded whether or not the dog showed any sign of stress. In the threatening approach test, we coded whether the dog showed any sign of fear or aggression just before the test was terminated (when the strongest threat was exposed to the dog, see **Table 1** for details). We used each of these binomial measures to categorize dogs into “stressed” and “non-stressed” groups based on their behavior in each of the *Greeting during separation* and *Greeting after threatening approach* tests.

#### Genotyping

We collected buccal samples from the dogs in a non-invasive manner by swabbing the upper gum area of the dogs with four cotton tips (see Héjjas et al., 2007). The cotton tips were then sealed in a tube and preserved in the freezer until genotyping.

The procedures of the DNA isolation, and sequencing and genotyping the SNPs were the same as described in Bence et al. (2017). The -213A/G, -74C/G, and the rs8679682 polymorphisms were genotyped by the PCR-RFLP method. PCR amplification was performed using 5′-9-CCA TTG GAA TCC GCC CCC T-3′-9 forward and 5′-9-CAC CAC CAG GTC GGC TAT G-3′9 reverse primers for -213AG and -74CG SNPs and 5′-GAA AGG CCA TTC TCA GGA AA-3′ forward and 5′-CCC CCA TCA TCT TCT ACC A-3′ reverse primers for rs8679682 SNP. Annealing temperature was 56°C and the total reaction volume was 10 ml. The PCR products were incubated for 3 h at 37°C in a restriction enzyme mixture containing 0.5 U/μl Hpy99I restriction enzyme (NEB) for -213A/G SNP, 0.5 U/μl BsiEI restriction enzyme (NEB) for -74C/G SNP, and 0.5 U/μl PshAi restriction enzyme (NEB) for rs8679682 SNP with 1× BSA and 1× NEB4 buffer. The -94T/C SNP was genotyped by allele-specific amplification (ASA) using the primers described above. Allele-specific primers were 5′-CCG ATC TGC TGG TCC CGG-3′ and 5′-CCG ATC TGC TGG TCC CGA-3′ and the annealing temperature was 60°C. The digested PCR products were analyzed by conventional submarine agarose gel electrophoresis (Biocenter, Szeged, Hungary), using 2.5% agarose gel and visualized by ethidium bromide staining.

Of the eight SNPs found in the *OXTR* gene in dogs, only four were polymorph enough (i.e., both homozygotes were present) in Border collies to be included in the current study. The genotype frequencies and the results of the Hardy–Weinberg equilibrium analyses of these four polymorphisms are shown in **Table 3**. In two cases (-213A/G and the rs8679682 SNPs), the rare homozygotes were <15%, therefore we combined them with the heterozygotes.

**Table 3:** Genotype frequencies and Hardy–Weinberg analyses of the four *OXTR* SNPs analyzed in this study.

Polymorphism	Genotype	*N*	%	χ^2^ test for HWE violation
-213A/G	AA	9	5.2	*p* = 0.539
	AG	55	31.8	
	GG	19	63.0	
-94T/C	CC	30	17.3	*p* = 0.013
	CT	102	59.0	
	TT	41	23.7	
-74C/G	CC	39	22.5	*p* = 0.001
	CG	51	29.5	
	GG	83	48.0	
rs8679682	CC	25	14.5	*p* = 0.060
	CT	97	56.1	
	TT	51	29.5	


*Strong statistical significance (*p* < 0.00001) would suggest that a given SNP may be subjected to disturbing factors (e.g., mutation, selection, nonrandom mating, genetic drift, or gene flow), which may bias the results of gene–behavior association analyses.*

### Statistical Analyses

First, in order to provide descriptive analyses, we investigated if the four possible modifying factors: context, sex, age, and stress in the preceding situation *per se* have a significant effect on the greeting behavior of the dogs. The context, sex, and age were included in a generalized linear mixed model as main effects, with the greeting behavior as the dependent variable and the dogs’ ID as a random effect. The model also included all two-way and three-way interactions. The effect of being stressed or not in the preceding situation was investigated with independent *t*-tests in the *Greeting during separation* and *Greeting after threatening approach* contexts separately. Next, to investigate whether there was any association between our subjects’ different individual characteristics (genotype distributions of the four SNPs, stress during separation, reaction to threatening, and sex of the dogs), we analyzed the relationships between these variables using χ^2^ tests.

Second, we used another generalized linear mixed model to analyze if the dogs’ individual characteristics (sex, age) and/or the context modified the association between the dogs’ *OXTR* genotype and behavior. This model included the greeting behavior as the dependent variable, the four SNPs, sex, and context as fixed factors, age as covariate, and the dogs’ ID as random effect. Here we also included all age × SNP, sex × SNP, and context × SNP interactions, as well as all sex × SNP × context and age × SNP × context interactions. Non-significant effects were removed from the model using a backward elimination procedure.

Third, we ran an additional GLM for each of the *Greeting during separation* and *Greeting after threatening approach* tests, in order to investigate how the dogs’ reaction to the preceding situation moderated the association between the *OXTR* genotypes and the behavior. The dogs’ reaction to the preceding situation was not added as a fixed factor in the previous models because the expected two-way or three-way interactions between SNP and reaction (and sex or age) may not be detected due to the lower sample size (*N* = 73–100 dogs per stress category). Therefore, we analyzed the effect of the SNPs separately in the two reaction categories of dogs using GLM models with the same setup as described above. In all the models, the effect size of each factor was estimated with Partial η^2^. SPSS version 22 was used for the analyses.

## Results

### Descriptive Analyses

**FIGURE 1:**
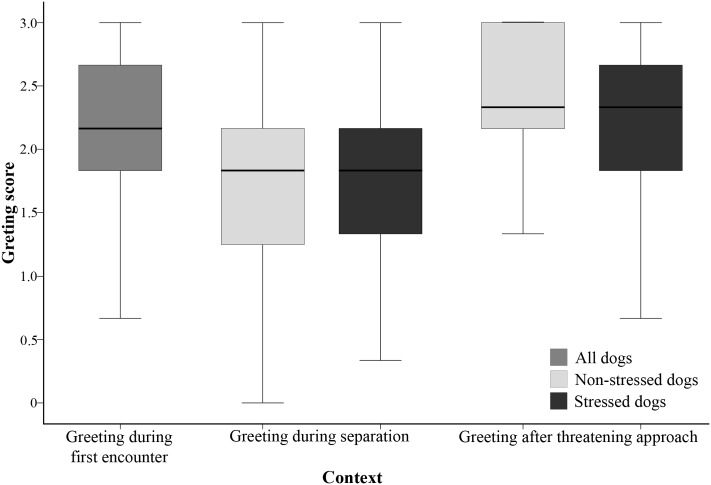
Greeting score of the dogs in the three contexts. Significant differences were found between the *Greeting during separation* and the other two contexts. For the *Greeting during separation* and *Greeting after threatening approach* contexts, the stressed and non-stressed dogs are presented separately, but no differences were found between the stressed and non-stressed dogs in any of these contexts.

Regarding the factors affecting the greeting behavior, neither the dogs’ sex, nor the age, nor any of their interactions were significantly related to the greeting behavior (*p* > 0.261 at removal). We found a main effect of the context (*F*_2,511_ = 50.760, *p* < 0.001), pairwise contrast revealed that the dogs greeted the experimenter more when they first met her and after threatening approach than during separation (*p* < 0.001 for both), but there was no difference between the *Greeting during first encounter* and *Greeting after threatening approach* contexts (*p* = 0.728) (**Figure 1**).

We found no significant differences between the stressed and non-stressed dogs either in the *Greeting during separation* or in the *Greeting after threatening approach* contexts (*p* = 0.206; *p* = 0.289, respectively) (**Figure 1**).

Regarding possible correlated effects of the different individual characteristics, we found no significant associations between stress during separation, reaction to the threatening approach, sex of the dog, and the genotype distributions of the four SNPs (**Table 4**).

**Table 4:** Relationship between genotype distributions, stress during separation, reaction to threatening, and the dogs’ sex.

	Sex			Stress during separation			Reaction to threatening		
			
	χ^2^	*df*	*p*	χ^2^	*df*	*p*	χ^2^	*df*	*p*
Stress during separation	0.206	1	0.650	-	-	-	0.228	1	0.633
Reaction to threatening	0.553	1	0.457	0.228	1	0.633	-	-	-
-213A/G	3.242	1	0.072	0.426	1	0.514	1.662	1	0.203
rs8679682	0.567	1	0.452	0.849	1	0.357	0.026	1	0.871
-94T/C	0.679	2	0.712	0.238	2	0.888	2.469	2	0.291
-74C/G	2.017	2	0.365	3.074	2	0.215	1.094	2	0.579


### Overall Analyses of *OXTR* and Context Effects

We found two significant sex × SNP × context and two significant age × SNP × context interactions, as well as a significant two-way interaction between SNP and context. The -213A/G SNP showed a significant three-way interaction with age and context (*F*_2,464_ = 3.151, *p* = 0.044), the -94T/C SNP with sex and context (*F*_4,464_ = 3.174, *p* = 0.014), and the -74C/G with both age and context and sex and context (*F*_4,464_ = 3.798, *p* = 0.005; *F*_4,464_ = 2.427, *p* = 0.047, respectively). The rs8679682 SNP had no interaction with sex or age, but we found a two-way interaction with context (*F*_2,464_ = 4.361, *p* = 0.013): the difference between this SNP’s genotypes was larger in the *Greeting during separation* context than in the other two contexts. To further explore and interpret the three-way interactions, we also analyzed the three contexts separately using general linear models (GLM), including the four SNPs, sex, age, and all age × SNP and sex × SNP interactions. Pairwise contrast was used for *post hoc* tests for main effects and sex interactions; the significant age interactions were interpreted by investigating the effect of age in the different genotypes separately.

### Context-Specific Analyses

#### Greeting during First Encounter

**FIGURE 2:**
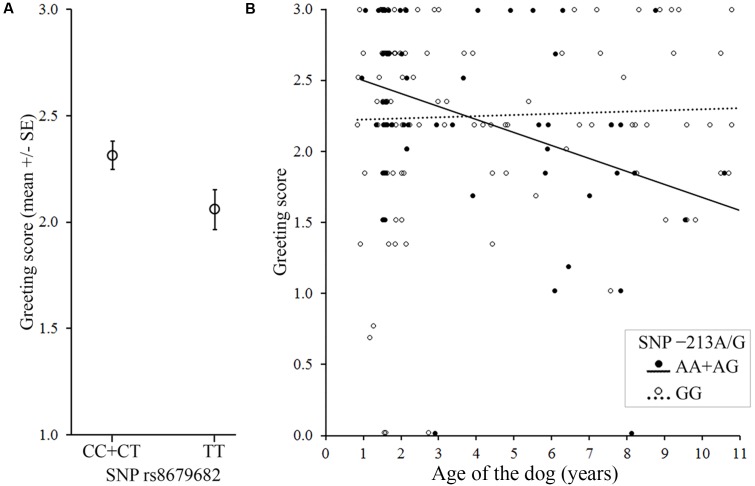
Associations between the *OXTR* SNPs and the behavior score in the Greeting during first encounter. **(A)** Dogs carrying the C allele in the rs8679682 SNP greeted the experimenter more than dogs with TT genotype. **(B)** Older dogs carrying the A allele in the -213A/G SNP greeted the experimenter less than younger dogs, while no age effect was found in GG genotype.

We found a significant main effect of the rs8679682 SNP (Partial η^2^ = 0.026), and an interaction between -213A/G SNP and age (Partial η^2^ = 0.033) (**Table 5** and **Figure 2**).

**Table 5:** The effects of dog *OXTR* polymorphisms and dog characteristics on behavior in the *Greeting during first encounter* context.

Source	*df*	*F*	*p*	Partial η^2^	*Post hoc* comparisons
Corrected model	4	3.339	0.012	0.074	
rs8679682	1	4.422	0.037	0.026	CC+CT > TT
-213A/G	1	6.262	0.013	0.036	
Age	1	4.325	0.039	0.025	
-213A/G × age	1	5.695	0.018	0.033	AA+AG: younger > older (*p* = 0.006); GG: no age effect (*p* = 0.841)
Total	171				


#### Greeting during Separation

**FIGURE 3:**
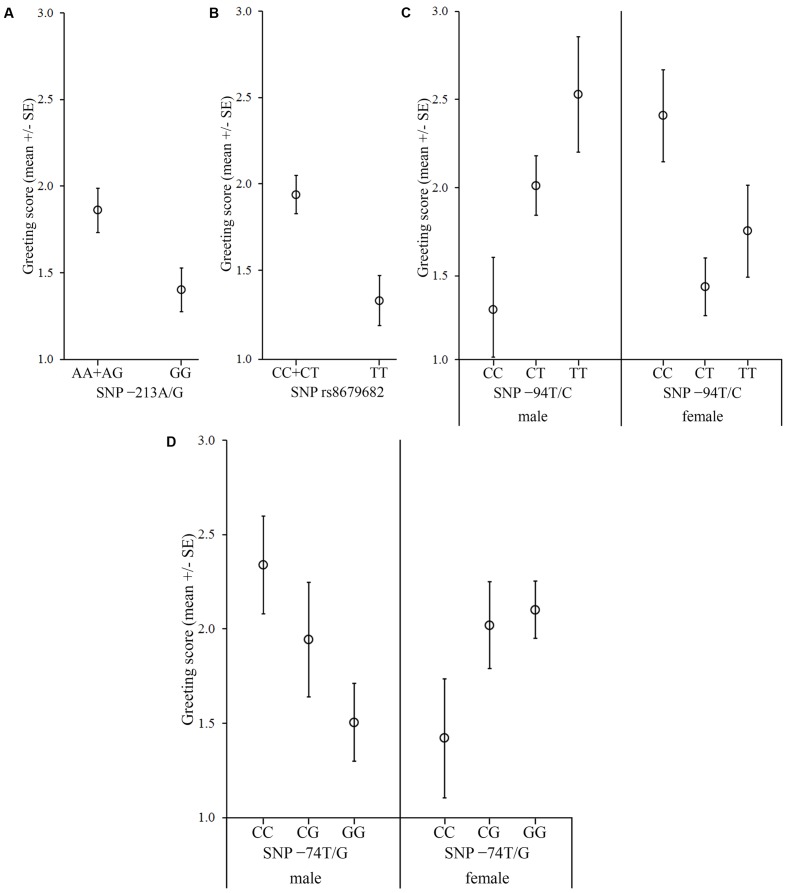
Associations between the *OXTR* SNPs and the behavior score in the Greeting during separation. The dogs were divided into two groups based on the behavior during separation. In the case of dogs, which did not show stress signals during separation, **(A)** dogs carrying the A allele in the -213A/G SNP greeted the experimenter more than dogs carrying the GG genotype, and **(B)** dogs carrying the C allele in the rs8679682 SNP greeted the experimenter more than dogs with a TT genotype. In the case of dogs, which showed stress signals during separation, **(C)** females carrying CC genotype in the -94T/C SNP greeted the experimenter more than the CT and TT genotypes, while the relation was the opposite in males; **(D)** females carrying the GG genotype in the -74C/G SNP received higher scores than those carrying the CC genotype, while the relation was the opposite in males.

We found significant main effects of the -213A/G and rs8679682 SNPs (Partial η^2^ = 0.030 and 0.076, respectively), and significant sex interactions of the -94T/C and -74C/G SNPs (Partial η^2^ = 0.060 and 0.054, respectively) (**Table 6a** and **Figure 3**).

**Table 6:** The effects of dog *OXTR* polymorphisms and dog characteristics on behavior in the *Greeting during separation* context.

Source	*df*	*F*	Sig.	Partial η^2^	*Post hoc* comparisons
**(a) All dogs**					
Corrected model	11	2.455	0.007	0.147	
Sex	1	0.052	0.820	0.000	
-213A/G	1	4.877	0.029	0.030	AA+AG > GG
-94T/C	2	0.327	0.722	0.004	
-74C/G	2	0.714	0.491	0.009	
rs8679682	1	12.892	0.000	0.076	CC+CT > TT
Sex ×-94T/C	2	4.968	0.008	0.060	females: CC > CT, TT (*p* = 0.019, *p* = 0.018); males: TT > CC (*p* = 0.046)
Sex ×-74C/G	2	4.522	0.012	0.054	females: GG > CG, CC (*p* = 0.018, *p* = 0.021); males CC ∼> GG (*p* = 0.064)
Total	169				

**(b) Only stressed dogs**					
Corrected model	9	2.095	0.041	0.205	
Sex	1	0.168	0.683	0.002	
-94T/C	2	1.743	0.182	0.046	
-74C/G	2	0.315	0.731	0.009	
Sex ×-94T/C	2	6.692	0.002	0.155	females: CC > CT, TT (*p* = 0.004, *p* = 0.086); males: CT, TT > CC (*p* = 0.046, *p* = 0.011)
Sex ×-74C/G	2	4.231	0.018	0.104	females: GG ∼> CC (*p* = 0.074); males: CC > GG (*p* = 0.024)
Total	83				

**(c) Only non-stressed dogs**					
Corrected model	2	5.333	0.007	0.114	
-213A/G	1	5.866	0.018	0.066	AA+AG > GG
rs8679682	1	9.738	0.002	0.105	CC+CT > TT
Total	86				


*(a) All dogs were analyzed; (b) only dogs that showed stress signals during separation were analyzed; (c) only dogs that did not show stress signals during separation were analyzed.*

In this context, we also investigated if the dogs’ reaction to separation moderated the associations between the SNPs and behavior. In the dogs that showed stress signals during separation, we found significant sex interactions of the -94T/C and -74C/G SNPs (Partial η^2^ = 0.155 and 0.104, respectively), but no main effects of the other two SNPs (*p* > 0.180 at removal) (**Table 6b**). In the dogs that did not show stress signals during separation, we found significant main effects of the -213A/G and rs8679682 SNPs (Partial η^2^ = 0.066 and 0.105, respectively), but no sex interactions of the other two SNPs (*p* > 0.501 at removal) (**Table 6c**).

#### Greeting after Threatening Approach

**FIGURE 4:**
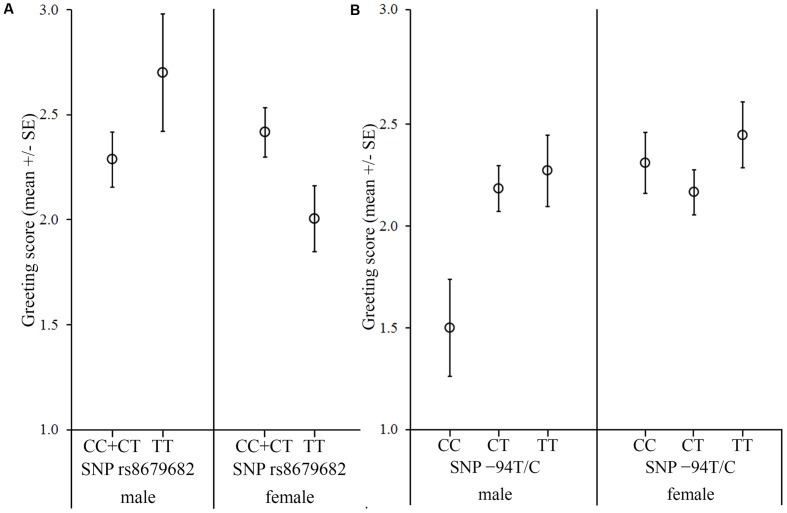
Associations between the *OXTR* SNPs and the behavior score in the Greeting after threatening approach. The dogs were divided into two groups based on the behavior during the threatening approach. **(A)** In the case of dogs, which reacted with friendly or passive behaviors to the threatening approach, we found a significant sex interaction of the rs8679682 SNP female dogs carrying the C allele greeted the experimenter more than females with TT genotype, while no genotype difference was found in male dogs. **(B)** In the case of dogs, which reacted with avoidance or aggression to the threatening approach, we found a sex interaction of the -94T/C SNP; males carrying the CC genotype greeted the experimenter less than the CT and TT genotypes, while no genotype difference was found in females.

We found no significant main effects or interactions of any SNPs (*p* > 0.141 at removal) (**Table 7a** and **Figure 4**). In this context, we also investigated if the dogs’ reaction to the threatening approach moderated the associations between the SNPs and behavior. In the dogs that reacted with avoidance or aggression to the threatening approach, we found a significant sex interaction of the -94T/C SNP (Partial η^2^ = 0.066) (**Table 7b**). In the dogs that reacted with friendly or passive behaviors to the threatening approach, we found a significant sex interaction of the rs8679682 SNP (Partial η^2^ = 0.070) (**Table 7c**). A summary of the results of the different models can be found in **Table 8**.

**Table 7:** The effects of dog *OXTR* polymorphisms and dog characteristics on behavior in the *Greeting after threatening approach* context.

Source	*df*	*F*	Sig.	Partial η^2^	*Post hoc* comparisons
**(a) All dogs**					
No significant effect					

**(b) Only stressed dogs**					
Corrected model	5	2.377	0.045	0.112	
Sex	1	5.847	0.018	0.059	
-94T/C	2	3.052	0.052	0.061	
Sex ×-94T/C	2	3.321	0.040	0.066	females: no difference; males: CT, TT > CC (*p* = 0.011, *p* = 0.010)
Total	100				

**(c) Only non-stressed dogs**					
Corrected model	3	2.209	0.095	0.089	
Sex	1	2.398	0.126	0.034	
rs8679682	1	0.000	0.995	0.000	
Sex × rs8679682	1	5.128	0.027	0.070	females: CC+CT > TT (*p* = 0.039); males: no difference
Total	72				


*(a) All dogs were analyzed; (b) only stressed dogs (which reacted with avoidance or aggression to the threatening approach) were analyzed; (c) only non-stressed dogs (which reacted with friendly or passive behaviors to the threatening approach) were analyzed.*

**Table 8:** Overview of the *OXTR* polymorphism–greeting associations found in the three contexts.

		*OXTR* polymorphism			
		
Context	Sample of dogs	-213A/G	rs8679682	-94T/C	-74C/G
First encounter	All dogs	AA+AG > GG	CC+CT > TT	–	–
In absence of owner	Without separation stress	AA+AG > GG	CC+CT > TT	–	–
	With separation stress	–	–	♀: CC > CT, TT	♀: GG > CC
				♂: CT, TT > CC	♂: CC > GG
After threat	Passive or friendly	–	♀: CC+CT > TT	–	–
			♂: –		
	Avoidant or aggressive	–	–	♀: –	–
				♂: CT, TT > CC	


## Discussion

Our goal in this study was to investigate how the dogs’ oxytocin system interacts with the context of the situation, the stress reactivity of the dogs and their other individual characteristics to predict social approach and affiliative behavior in social encounters. To analyze this, we examined four polymorphisms of the dogs’ *OXTR* gene, assessed the dogs’ greeting behavior in three different contexts, and grouped the dogs based on their reaction to separation and social threat, respectively. Overall, we found that similarly to previous studies (see reviewed in Kis et al., 2017) all four *OXTR* polymorphisms were significantly associated with greeting in at least one context. However, we also showed that both the context of the greeting and the dogs’ being stressed had a moderating effect on these associations.

### Context and Stress in Association with Greeting Behavior

In the first context, upon their *first encounter*, the dogs had the chance to greet the unfamiliar experimenter in the presence of their owner. In the *Greeting after threatening approach* context, the dogs greeted the more or less familiar experimenter also in the presence of the owner; however, in this context the dogs had a clearly negative experience with the experimenter immediately before the greeting situation. Although based on this we expected that dogs would greet the experimenter less enthusiastically after being threatened than during their first encounter, we found that the dogs greeted the experimenter with a similar intensity in these two contexts. This likely reflects the fact that dogs can flexibly adjust their behavior to the behavior (attitude) of their human partners (as Vas et al., 2005 also showed). This may also explain why no difference was found between the greeting behavior of dogs that had responded with or without overt stress to the social threat (i.e., dogs that showed avoidance or aggression vs. behaved in a friendly manner or remained passive). In contrast to the two contexts above, in the *Greeting during separation* context, the owner was not present during the test, and in this context the dogs greeted the experimenter less intensively than in the other two contexts. Although some of the dogs showed clear signs of stress caused by being separated from their owner and the others did not, the greeting intensity of both groups was uniformly lower in this context than in the presence of the owner. This suggests that the presence of the owner buffers the stress caused by the test situation, probably by providing social support (Gácsi et al., 2013).

Approximately half of the dogs (48.5%) showed stress-related behaviors during separation, and 57.8% of the dogs reacted with avoidance or aggression to the threatening approach (categorized as “stressed” based on Gácsi et al., 2013 and Horváth et al., 2008). It is important to note that we found no association between dogs’ signs of stress in response to separation and their behavior in response to a social threat. This means that the stressed vs. non-stressed categorization of the dogs did not reflect their general stress proneness, or willingness to show overt behavioral expression of their stress. Instead, showing behaviors related to stress seems to indicate a negative reaction specific to either test context: either separation anxiety or fear of a threatening person.

### *OXTR* Polymorphism–Behavior Associations

The most important finding of this study is that different *OXTR* SNPs were associated with greeting in stressed dogs and non-stressed dogs in either context. When trying to interpret these results, we should first mention that neither the separation stress nor the reaction to threatening was significantly related to any SNPs (or to the sex of the dog). Therefore, despite numerous studies suggesting the contrary (e.g., Kumsta and Heinrichs, 2013; Buttner, 2016), we cannot conclude that the *OXTR* polymorphisms themselves were directly related to general stress reduction or lower perception of social fear – at least not indiscriminately. Instead, the different SNPs had different behavioral associations depending on how the dog reacted to the context. As a possible explanation, stressed dogs may have approached and greeted the experimenter for different reasons than non-stressed dogs; even if they greeted her to a similar extent, they may have had different motivations to do so. This explanation would indicate that the dogs that did not show a negative reaction to separation or to a social threat maintained the same motivation to greet the experimenter as they had during the first encounter. However, for the dogs that were stressed by either being separated from their owner or threatened, the motivation to affiliate with the experimenter may have changed due to separation stress or social fear. For example, during separation, stressed dogs might look more for social support from the experimenter than non-stressed dogs, as they are more strongly affected by being alone.

In the context of the *Greeting during separation*, the -94T/C and -74C/G SNPs were found in association with the greeting only in stressed dogs, and both had a sex interaction. For the -94T/C, stressed males carrying the TT genotype greeted the experimenter more than males with the CC genotype, while the relation was opposite or absent in female dogs. For the -74C/G, stressed males carrying the CC genotype greeted the experimenter more than males carrying the GG genotype, while the relation was the opposite in the case of females. Human studies also found not only stress-dependent *OXTR*–behavior associations, as described in the section “Introduction,” but also sex differences in the effects of social support on the stress response (i.e., social support attenuated the stress reaction more for men than women, Kirschbaum et al., 1995; Ditzen et al., 2008).

Interestingly, even though the separation from the owner and a threatening person presented different types of stress for the dogs, the -94T/C SNP had a similar association with the greeting behavior also in the *Greeting after threatening approach* context. That is, stressed males carrying the TT genotype greeted the experimenter more than males with the CC genotype both when their owners left them alone, and when the experimenter had successfully threatened them beforehand. This seems to indicate that the -94T/C has a more general function in regulating the behavior of stressed (especially male) individuals, but, whether this SNP regulates the social fear of stressed dogs or affects their general stress coping ability should be further investigated.

In contrast, the polymorphisms -213A/G and rs8679682 were associated with greeting in non-stressed dogs during separation, and, in the case of the rs8679682, also after being threatened. Importantly, these same two SNPs were associated with the behavior also in the *Greeting during first encounter* context. This seems to indicate that dogs in general did not perceive the first encounter with the experimenter as stressful.

For the -213A/G, individuals carrying the A allele showed more intense greeting than dogs with the GG genotype. Since this SNP was associated with the dogs’ behavior only when the experimenter presented a positive or neutral figure (i.e., *Greeting during first encounter* and *Greeting during separation* in non-stressed dogs), the effect of this SNP might be sensitive to the characteristics of the experimenter. That is, the A allele predicts increased social approach and affiliation but only when the dog had no prior negative experience with the experimenter. Similarly, human studies also found differential effects of oxytocin on social behavior depending on the attitude of the social partner (e.g., Mikolajczak et al., 2010; De Dreu, 2012). Moreover, Kis et al. (2014) also found significant associations between the -213A/G and dogs’ proximity seeking (a behavior scale that includes variables assessed in different greeting situations) both in German shepherds and in Border collies, and similarly to our study, they found that in Border collies, carrying the A allele was associated with higher proximity seeking.

For the rs8679682 SNP, non-stressed dogs carrying the C allele showed more intense greeting than dogs with the TT genotype in all three contexts (however, this association might be stronger in females than in males). This seems to indicate that the rs8679682 has a more general function in regulating the social affiliative behavior of dogs toward humans (with the C allele facilitating more positive social behaviors relative to the T allele), but its effects seem to be sensitive to the positive salience of the context. Whether this SNP regulates social motivation, attraction to humans or general curiosity needs further investigation.

Another parallel between our results and other studies is that the sex of the dog also modulates the gene × behavior associations. Sex differences in the function of the oxytocin system are well known (Bos et al., 2012; Chen and Johnson, 2012; Kovács et al., 2016), and can be explained by possible differences in the general hormonal environment (e.g., estrogen level, McCarthy, 1995; Petersson et al., 1999), different patterns of oxytocin release (Jezová et al., 1996), and/or different patterns of amygdala activation between the sexes. For example, oxytocin administration decreases amygdala reactivity to emotional faces in men (Domes et al., 2007) and increases it in women (Domes et al., 2010). Our results suggest that also in dogs, some relationships between polymorphisms of the *OXTR* and social behavior seem to be sex-specific.

## Conclusion

Taken together, our results show that dogs’ greeting behavior depends on the interaction of three factors: the context of the greeting, the individual characteristics of the animal (stress level and sex), and the genotype the dog carries in a given *OXTR* polymorphism. These results seem to be (at least partly) in harmony with the social salience hypothesis suggested in human studies (e.g., Shamay-Tsoory and Abu-Akel, 2016). That is, the way the *OXTR* polymorphisms are associated with behavior seems to depend on how the dogs perceived the situation itself, which, in turn, depends both on the context and the individual inclinations of the dogs.

These results can serve as a starting point for follow-up studies potentially demonstrating that much of the variance observed in the behavioral associations of the *OXTR* polymorphisms is systematic and a function of the context-, stress-, and individual-dependent nature of the effects of *OXTR* variation. In humans, consistent results of multiple studies employing similar procedures have been used to form such a conclusion, and to infer about the psychological and/or biological processes at play (Bartz et al., 2011). Based on our results, we suggest that the four polymorphisms investigated here might be related to different functions of the oxytocin system, as each of them was associated with behavior only in either positively or in negatively perceived situations. Beyond this, the functionality of these SNP variants is still unclear (especially because three of our SNPs are located in non-translating regions), so further molecular studies are warranted to elucidate functional consequences of these variants.

## Author Contributions

BT, FR, ZR, and ZV designed the study. BT and DK acquired and analyzed the data. BT, FR, and ZV interpreted the data. ZV, FR, ZR, and BT obtained funding. BT and DK wrote the first draft of the manuscript. All authors critically revised the manuscript for important intellectual content. All authors gave final approval of the manuscript version to be published and agreed to be accountable for all aspects of the work in ensuring that questions related to the accuracy or integrity of any part of the work are appropriately investigated and resolved.

## Conflict of Interest Statement

The authors declare that the research was conducted in the absence of any commercial or financial relationships that could be construed as a potential conflict of interest. The reviewer MM and handling Editor declared their shared affiliation.

## Footnotes

1 http://www.vetmeduni.ac.at/fileadmin/v/z/forschung/GoodScientificPractice_English.pdf

## References

[B1] Bartz J. A. Zaki J. Bolger N. Ochsner K. N. (2011). Social effects of oxytocin in humans: context and person matter. *Trends Cogn. Sci.* 15 301–309. 10.1016/j.tics.2011.05.002 21696997

[B2] Beetz A. Uvnäs-Moberg K. Julius H. Kotrschal K. (2012). Psychosocial and psychophysiological effects of human-animal interactions: the possible role of oxytocin. *Front. Psychol.* 3:234. 10.3389/fpsyg.2012.00234 22866043 PMC3408111

[B3] Bence M. Marx P. Szantai E. Kubinyi E. Ronai Z. Banlaki Z. (2017). Lessons from the canine *Oxtr* gene: populations, variants and functional aspects. *Genes Brain Behav.* 16 427–438. 10.1111/gbb.12356 27860243

[B4] Benjamin D. J. Cesarini D. van der Loos M. J. Dawes C. T. Koellinger P. D. Magnusson P. K. (2012). The genetic architecture of economic and political preferences. *Proc. Natl. Acad. Sci. U.S.A.* 109 8026–8031. 10.1073/pnas.1120666109 22566634 PMC3361436

[B5] Bos P. A. Panksepp J. Bluthé R. M. van Honk J. (2012). Acute effects of steroid hormones and neuropeptides on human social–emotional behavior: a review of single administration studies. *Front. Neuroendocrinol.* 33 17–35. 10.1016/j.yfrne.2011.01.002 21256859

[B6] Buttner A. P. (2016). Neurobiological underpinnings of dogs’ human-like social competence: how interactions between stress response systems and oxytocin mediate dogs’ social skills. *Neurosci. Biobehav. Rev.* 71 198–214. 10.1016/j.neubiorev.2016.08.029 27593441

[B7] Campbell A. (2008). Attachment, aggression and affiliation: the role of oxytocin in female social behavior. *Biol. Psychol.* 77 1–10. 10.1016/j.biopsycho.2007.09.001 17931766

[B8] Chen F. S. Johnson S. C. (2012). An oxytocin receptor gene variant predicts attachment anxiety in females and autism-spectrum traits in males. *Soc. Psychol. Personal. Sci.* 3 93–99. 10.1177/1948550611410325

[B9] Chen F. S. Kumsta R. von Dawans B. Monakhov M. Ebstein R. P. Heinrichs M. (2011). Common oxytocin receptor gene (*OXTR*) polymorphism and social support interact to reduce stress in humans. *Proc. Natl. Acad. Sci. U.S.A.* 108 19937–19942. 10.1073/pnas.1113079108 22123970 PMC3250137

[B10] De Dreu C. K. (2012). Oxytocin modulates cooperation within and competition between groups: an integrative review and research agenda. *Horm. Behav.* 61 419–428. 10.1016/j.yhbeh.2011.12.009 22227278

[B11] Depue R. A. Morrone-Strupinsky J. V. (2005). A neurobehavioral model of affiliative bonding: implications for conceptualizing a human trait of affiliation. *Behav. Brain Sci.* 28 313–349. 10.1017/S0140525X05280068 16209725

[B12] Ditzen B. Schmidt S. Strauss B. Nater U. M. Ehlert U. Heinrichs M. (2008). Adult attachment and social support interact to reduce psychological but not cortisol responses to stress. *J. Psychosom. Res.* 64 479–486. 10.1016/j.jpsychores.2007.11.011 18440400

[B13] Domes G. Heinrichs M. Gläscher J. Büchel C. Braus D. F. Herpertz S. C. (2007). Oxytocin attenuates amygdala responses to emotional faces regardless of valence. *Biol. Psychiatry* 62 1187–1190. 10.1016/j.biopsych.2007.03.025 17617382

[B14] Domes G. Lischke A. Berger C. Grossmann A. Hauenstein K. Heinrichs M. (2010). Effects of intranasal oxytocin on emotional face processing in women. *Psychoneuroendocrinology* 35 83–93. 10.1016/j.psyneuen.2009.06.016 19632787

[B15] Donaldson Z. R. Young L. J. (2008). Oxytocin, vasopressin, and the neurogenetics of sociality. *Science* 322 900–904. 10.1126/science.1158668 18988842

[B16] Eiji Nawa N. Nelson E. E. Pine D. S. Ernst M. (2008). Do you make a difference? Social context in a betting task. *Soc. Cogn. Affect. Neurosci.* 3 367–376. 10.1093/scan/nsn032 19015081 PMC2607052

[B17] Gácsi M. Maros K. Sernkvist S. Faragó T. Miklósi Á. (2013). Human analogue safe haven effect of the owner: behavioural and heart rate response to stressful social stimuli in dogs. *PLOS ONE* 8:e58475. 10.1371/journal.pone.0058475 23469283 PMC3587610

[B18] Héjjas K. Kubinyi E. Rónai Z. Székely A. Vas J. Miklósi Á. (2009). Molecular and behavioral analysis of the intron 2 repeat polymorphism in the canine dopamine D4 receptor gene. *Genes Brain Behav.* 8 330–336. 10.1111/j.1601-183X.2008.00475.x 19382953

[B19] Héjjas K. Vas J. Topál J. Szántai E. Rónai Z. Székely A. (2007). Association of polymorphisms in the dopamine D4 receptor gene and the activity-impulsivity endophenotype in dogs. *Anim. Genet.* 38 629–633. 10.1111/j.1365-2052.2007.01657.x 17986156

[B20] Horváth Z. Dóka A. Miklósi Á. (2008). Affiliative and disciplinary behavior of human handlers during play with their dog affects cortisol concentrations in opposite directions. *Horm. Behav.* 54 107–114. 10.1016/j.yhbeh.2008.02.002 18353328

[B21] Insel T. R. (2003). Is social attachment an addictive disorder? *Physiol. Behav.* 79 351–357. 10.1016/S0031-9384(03)00148-3 12954430

[B22] Insel T. R. Shapiro L. E. (1992). Oxytocin receptor distribution reflects social organization in monogamous and polygamous voles. *Proc. Natl. Acad. Sci. U.S.A.* 89 5981–5985. 10.1073/pnas.89.13.5981 1321430 PMC402122

[B23] Jezová D. Juránková E. Mosnárová A. Kriška M. (1996). Neuroendocrine response during stress with relation to gender differences. *Acta Neurobiol. Exp.* 56 779–785. 10.55782/ane-1996-1183 8917906

[B24] Kim H. S. Sherman D. K. Sasaki J. Y. Xu J. Chu T. Q. Ryu C. (2010). Culture, distress, and oxytocin receptor polymorphism (OXTR) interact to influence emotional support seeking. *Proc. Natl. Acad. Sci. U.S.A.* 107 15717–15721. 10.1073/pnas.1010830107 20724662 PMC2936623

[B25] Kirschbaum C. Klauer T. Filipp S. H. Hellhammer D. H. (1995). Sex-specific effects of social support on cortisol and subjective responses to acute psychological stress. *Psychosom. Med.* 57 23–31. 10.1097/00006842-199501000-00004 7732155

[B26] Kis A. Bence M. Lakatos G. Pergel E. Turcsán B. Pluijmakers J. (2014). Oxytocin receptor gene polymorphisms are associated with human directed social behavior in dogs (*Canis familiaris*). *PLOS ONE* 9:e83993. 10.1371/journal.pone.0083993 24454713 PMC3893090

[B27] Kis A. Ciobica A. Topál J. (2017). The effect of oxytocin on human-directed social behaviour in dogs (*Canis familiaris*). *Horm. Behav.* 94 40–52. 10.1016/j.yhbeh.2017.06.001 28624235

[B28] Kovács K. Kis A. Pogány Á. Koller D. Topál J. (2016). Differential effects of oxytocin on social sensitivity in two distinct breeds of dogs (*Canis familiaris*). *Psychoneuroendocrinology* 74 212–220. 10.1016/j.psyneuen.2016.09.010 27665081

[B29] Kubinyi E. Bence M. Koller D. Wan M. Pergel E. Ronai Z. (2017). Oxytocin and opioid receptor gene polymorphisms associated with greeting behavior in dogs. *Front. Psychol.* 8:1520. 10.3389/fpsyg.2017.01520 28936190 PMC5594098

[B30] Kumsta R. Heinrichs M. (2013). Oxytocin, stress and social behavior: neurogenetics of the human oxytocin system. *Curr. Opin. Neurobiol.* 23 11–16. 10.1016/j.conb.2012.09.004 23040540

[B31] Li J. Zhao Y. Li R. Broster L. S. Zhou C. Yang S. (2015). Association of oxytocin receptor gene (OXTR) rs53576 polymorphism with sociality: a meta-analysis. *PLOS ONE* 10:e0131820. 10.1371/journal.pone.0131820 26121678 PMC4488068

[B32] Lim M. M. Young L. J. (2006). Neuropeptidergic regulation of affiliative behavior and social bonding in animals. *Horm. Behav.* 50 506–517. 10.1016/j.yhbeh.2006.06.028 16890230

[B33] MacDonald K. MacDonald T. M. (2010). The peptide that binds: a systematic review of oxytocin and its prosocial effects in humans. *Harv. Rev. Psychiatry* 18 1–21. 10.3109/10673220903523615 20047458

[B34] Maros K. Dóka A. Miklósi Á. (2008). Behavioural correlation of heart rate changes in family dogs. *Appl. Anim. Behav. Sci.* 109 329–341. 10.1016/j.applanim.2007.03.005

[B35] McCarthy M. M. (1995). Estrogen modulation of oxytocin and its relation to behavior. *Adv. Exp. Med. Biol.* 395 235–245. 8713972

[B36] Mehrkam L. R. Wynne C. D. (2014). Behavioral differences among breeds of domestic dogs (*Canis lupus familiaris*): current status of the science. *Appl. Anim. Behav. Sci.* 155 12–27. 10.1016/j.applanim.2014.03.005

[B37] Meyer-Lindenberg A. Domes G. Kirsch P. Heinrichs M. (2011). Oxytocin and vasopressin in the human brain: social neuropeptides for translational medicine. *Nat. Rev. Neurosci.* 12 524–538. 10.1038/nrn3044 21852800

[B38] Mikolajczak M. Gross J. J. Lane A. Corneille O. de Timary P. Luminet O. (2010). Oxytocin makes people trusting, not gullible. *Psychol. Sci.* 21 1072–1074. 10.1177/0956797610377343 20631321

[B39] Over H. (2016). The origins of belonging: social motivation in infants and young children. *Philos. Trans. R. Soc. B. Biol. Sci.* 371:20150072. 10.1098/rstb.2015.0072 26644591 PMC4685518

[B40] Palestrini C. Previde E. P. Spiezio C. Verga M. (2005). Heart rate and behavioural responses of dogs in the Ainsworth’s strange situation: a pilot study. *Appl. Anim. Behav. Sci.* 94 75–88. 10.1016/j.applanim.2005.02.005

[B41] Persson M. E. Roth L. S. V. Johnsson M. Wright D. Jensen P. (2015). Human-directed social behaviour in dogs shows significant heritability. *Genes Brain Behav.* 14 337–344. 10.1111/gbb.12194 25703740

[B42] Persson M. E. Wright D. Roth L. S. Batakis P. Jensen P. (2016). Genomic regions associated with interspecies communication in dogs contain genes related to human social disorders. *Sci. Rep.* 6:33439. 10.1038/srep33439 27685260 PMC5041581

[B43] Petersson M. Lundeberg T. Uvnäs-Moberg K. (1999). Short-term increase and long-term decrease of blood pressure in response to oxytocin-potentiating effect of female steroid hormones. *J. Cardiovasc. Pharmacol.* 33 102–108. 10.1097/00005344-199901000-00015 9890403

[B44] Shamay-Tsoory S. G. Abu-Akel A. (2016). The social salience hypothesis of oxytocin. *Biol. Psychiatry* 79 194–202. 10.1016/j.biopsych.2015.07.020 26321019

[B45] Svartberg K. Forkman B. (2002). Personality traits in the domestic dog (*Canis familiaris*). *Appl. Anim. Behav. Sci.* 79 133–155. 10.1016/S0168-1591(02)00121-1 28414470

[B46] Topál J. Miklósi Á. Csányi V. Dóka A. (1998). Attachment behavior in dogs (*Canis familiaris*): a new application of Ainsworth’s (1969) strange situation test. *J. Comp. Psychol.* 112 219–229. 10.1037/0735-7036.112.3.219 9770312

[B47] Tost H. Kolachana B. Hakimi S. Lemaitre H. Verchinski B. A. Mattay V. S. (2010). A common allele in the oxytocin receptor gene (*OXTR*) impacts prosocial temperament and human hypothalamic-limbic structure and function. *Proc. Natl. Acad. Sci. U.S.A.* 107 13936–13941. 10.1073/pnas.1003296107 20647384 PMC2922278

[B48] van der Waaij E. H. Wilsson E. Strandberg E. (2008). Genetic analysis of results of a Swedish behavior test on German shepherd dogs and labrador retrievers. *J. Anim. Sci.* 86 2853–2861. 10.2527/jas.2007-0616 18502884

[B49] Vas J. Topál J. Gácsi M. Miklósi A. Csányi V. (2005). A friend or an enemy? Dogs’ reaction to an unfamiliar person showing behavioural cues of threat and friendliness at different times. *Appl. Anim. Behav. Sci.* 94 99–115. 10.1016/j.applanim.2005.02.001

[B50] Vas J. Topál J. Gyõri B. Miklósi Á. (2008). Consistency of dogs’ reactions to threatening cues of an unfamiliar person. *Appl. Anim. Behav. Sci.* 112 331–344. 10.1016/j.applanim.2007.09.002

[B51] vonHoldt B. M. Shuldiner E. Koch I. J. Kartzinel R. Y. Hogan A. Brubaker L. (2017). Structural variants in genes associated with human Williams-Beuren syndrome underlie stereotypical hypersociability in domestic dogs. *Sci. Adv.* 3:e1700398. 10.1126/sciadv.1700398 28776031 PMC5517105

[B52] Wan M. Hejjas K. Ronai Z. Elek Z. Sasvari-Szekely M. Champagne F. A. (2013). *DRD4* and *TH* gene polymorphisms are associated with activity, impulsivity and inattention in siberian husky dogs. *Anim. Genet.* 44 717–727. 10.1111/age.12058 23713429

[B53] Weir J. P. (2005). Quantifying test-retest reliability using the intraclass correlation coefficient and the SEM. *J. Strength Cond. Res.* 19 231–240. 10.1519/15184.1 15705040

[B54] Young L. J. Lim M. M. Gingrich B. Insel T. R. (2001). Cellular mechanisms of social attachment. *Horm. Behav.* 40 133–138. 10.1006/hbeh.2001.1691 11534973

